# Association of CSN1S1 gene polymorphism on milk components of the Indonesian local PE cross-dairy goats

**DOI:** 10.5455/javar.2025.l889

**Published:** 2025-03-25

**Authors:** Ega Anggraini Ega, Cece Sumantri Cece, Afton Atabany Afton, Anneke Anggraeni Anneke, Tri Puji Priyatno

**Affiliations:** 1 Department of Animal Production and Technology, Faculty of Animal Science, Bogor Agriculture University, Bogor, Indonesia; 2 Research Center for Animal Husbandry, National Research and Innovation Agency, Bogor, Indonesia

**Keywords:** CSN1S1 gene, local dairy goat, milk component, SNPS

## Abstract

**Objective:**

This study aimed to identify Casein Alpha S1 (CSN1S1) gene polymorphism as a biomarker selection for improving the milk quality of Indonesian local PE cross-dairy goats.

**Materials and Methods:**

The study utilized 65 does to identify single nucleotide polymorphisms (SNPs) on the target base fragment g.10094 gm.10200 of the CSN1S1 gene. Milk components (MCs) were analyzed using lactoscan and SNPs were identified using Sanger sequencing. Allele and genotype frequencies of the SNPs were determined by MEGA10 and PopGen32 programs. A general linear model was applied to examine the association between each SNP and the content (%) or daily yield (gm/day) of each of the MCs.

**Results:**

Eight SNPs were identified, with seven exhibiting dominant homozygous genotype references with genotype frequencies ranging from 0.77 to 0.95. SNP g.10098_C > G significantly affected (*p *< 0.05) the daily yield (gm/day) of MCs, but not for TS. The CC does, compared to the GC ones, produce higher yields (gm/day) of protein, fat, lactose, and SNF by 36.2%–39.8%. SNP g.10181_T > A had a significant effect (*p *< 0.05) on the content (%) of all MCs. The AT does, over the AA and TT ones, yield higher MCs by 3.63%–13.07% and 1.85%–10.68%, respectively.

**Conclusio:**

The findings suggest that SNP g.10098_C > G and g.10181_T > A of the CSN1S1 gene may serve as potential biomarkers for selective breeding to enhance milk quality in the local PE cross-dairy goats.

## Introduction

Goat milk serves as a source of essential nutrients for humans because of the similarities between caprine and human milk. The bioactive compounds in milk provide numerous health benefits and yield nutritious processed products [1,2,3]. Many consumers prefer goat milk owing to its distinctive qualities, including flavor, smaller fat globules, specific coagulation properties, and reduced allergenicity compared to other milk types [4,5]. Protein is a component that determines milk quality, composition, and dairy products [2,4,6]. In ruminants, milk protein comprises two main fractions, namely whey, and casein, with casein constituting the largest proportion at more than 80% [2,7]. Casein protein is genetically encoded by the casein genes (250 kb) located on chromosome number six of the caprine genome. The CN gene cluster consists of CSN1S1, CSN2, CSN1S2, and CSN3, each of which functions in encoding the *α*s1-CN, *β*CN, *α*s2-CN, and *κ*-CN proteins [8–11]. Genetic variants of these four casein genes influence milk protein production, milk composition, cheese processing properties, digestibility, and tolerance in human nutrition [10].

Advances in molecular genetics offer numerous benefits, such as detecting individual genes or gene clusters that directly affect economically valuable traits. Casein genes are known to exhibit high genetic polymorphism [11,12]. The CSN1S1 gene consists of 19 exons ranging in size from 24 (exons 5, 6, 7, 8, 10, 13, and 16) to 385 bp (exon 19) and 18 introns ranging from 90 bp (intron 10) to 1,685 bp (intron 2) [2,13]. The CSN1S1 gene plays an important role in casein secretion [5]. The CSN1S1 protein constitutes approximately 47.21% of pure milk protein [4,14], thus contributing significantly to the structural characteristics, composition, nutrition, and milk products in goats [3].

The genetic mechanisms of major casein genes have been extensively investigated, particularly in European goat breeds [6,11,15]. Different breeds within the same species may exhibit distinct genetic responses to base mutations (SNPs) and their interactions within the same or different genes [11]. Some studies have also examined genetic polymorphisms of casein genes and their effects on milk protein and other milk components (MCs) in non-European goat breeds. Previous research on the CSN1S1 gene in these goats has uncovered significant genetic variability in the CSN1S1 gene and the important effects of their SNPs on the concentration of milk protein and other MCs [5,12,13,16].

Local PE cross-bred goats, also referred to as Sapera goats, are the crossbreds of the local PE (Peranakan Etawah/Etawah Grade) females with Saanen males. These crossbreds were developed to combine the high milk production of Saanen with the tropical adaptability of PE in Indonesia [15]. The local PE dairy-cross goats are intended to become a new breed with the advantages of high milk production and milk quality [16]. Enhancement of the contents and yields of milk protein and other MCs, as the objectives for improving milk quality, will possibly be implemented through molecular selection of the CSN1S1 gene. However, there is limited information regarding genetic polymorphisms (SNPs) and their effects on the milk protein and MCs of the Indonesian local PE goats and their crossbreeds. This study therefore aims to identify SNPs of the CSN1S1 gene and their favorable genotypes as potential SNPs that will be used as biomarker selection to improve milk quality in the local PE cross-dairy goats.

## Materials and Methods

### Ethical approval

The Animal Care and Use Committee of the Indonesian Agency of Agriculture Research and Development (IAARD), under the Indonesian Ministry of Agriculture, approved the protocols implemented in this investigation (Approval No. Balitbangtan/Balitnak/Rm/11/2021).

### Location and period of research

Animal observations, milk collection, and blood collection were conducted at the Dairy Goat Research Station of the Indonesian Research Institute for Animal Production (IRIAP), Ciawi, Bogor, West Java, Indonesia, from 2021 to 2023. The IRIAP is located 450–500 m above sea level in Banjar Waru Village, Ciawi Sub-district, Bogor Regency, and experiences an average annual precipitation of 3,500–4,000 mm/yr.

Milk components were analyzed at the Dairy Laboratory and Animal Product Technology Laboratory, whereas molecular analysis of CSN1S1 was conducted at the Animal Molecular Genetics Laboratory, Department of Animal Production and Technology, Faculty of Animal Science, Bogor Agriculture University, Bogor, West Java.

### Research samples

The research animals were the local PE dairy-cross goats, usually referred to as Sapera. These were crossbred offspring of local PE females and Saanen males, possessing a genetic composition of 50% PE and 50% Saanen. The research samples consisted of 65 does with lactation lengths ranging from 1 to ≥ 5 mo and lactation periods spanning from 1 to 4 mo within the observation period from 2021 to 2022.

The content and yield (gm/day) of milk protein and MCs of individual does were derived from a test of daily milk yields (DMYs), calculated as the sum of morning and noon production. Daily milk yield data were categorized according to lactation stage (1- ≥5), lactation period (1- ≥4), kidding season (January-March, April-June, July-September), and kidding year or initial year lactation (2021 and 2022).

The contents of milk protein and other MCs were determined using a Lactoscan device. The fundamental operational procedure involved placing a milk sample in the small tube of this apparatus, which was subsequently subjected to a sound wave beam to ascertain the contents of milk protein and other MCs. The daily yield (gm/day) of milk protein or other MCs was calculated using the following formula:

Protein yield (gm/day) = (DMY (ml/day) × specific gravity) × {(protein content (xgm) / (100 gm milk) × 10}

DMY: daily milk yield in milliliters per day (ml/day).

For deoxyribonucleic acid (DNA) analysis, approximately 3 ml of fresh blood was extracted from the jugular vein and transferred into a 10 ml volume tube containing ethylenediaminetetraacetic acid.

### Primary design

The primer sequence pairs used for amplification of the target base fragment of the CSN1S1 gene were custom-designed with the forward F: 5’-CTCATCCTCTGTCCTCTTCT-3’ and reverse R: 5’-CTGTGCTTTCACAAGGAGGC-3’. Multiplex PCR primers were designed for base positions 10178–10730 bp located on chromosome 6 in dairy goats. Primer design base sequence data were obtained from the National Center for Biotechnology Information with the accession code NC_030813.1 (https://www.ncbi.nlm.nih.gov/nuccore/NC_030813). The base length of the primers was determined using Primer 3 (http://www.primer3.org) and subsequently analyzed using Primer Stat (https://www.bioinformatics.org/sms2/pcr_primer _stats.html).

### DNA extraction

DNA was extracted utilizing a modified Geneaid DNA Kit (Geneaid Biotech Ltd., 2016). Blood samples (300 µl) were combined with 900 µl of the RBC lysis solution and homogenized. Following incubation at room temperature for 10 min, the sample was centrifuged at 3,000 rpm for 5 min, and the supernatant was discarded. The sample was subsequently mixed with 100 µl of RBC lysis solution and 200 µl of GB Buffer for homogenization, incubated at 60°C for 10 min, and inverted at 3 min intervals.

Subsequently, 5 µl of RNase was introduced, and the mixture was incubated at room temperature for 5 min. Following the addition of 200 µl absolute ethanol, the mixture was centrifuged at 16,000 rpm for 5 min. The supernatant was discarded, 400 µl of W1 buffer solution was added, and the mixture was centrifuged for 1 min. Diluted wash buffer (600 µl) was added, and the mixture was centrifuged at 16,000 rpm for 3 sec. A sample from the GD column was combined with 100 µl elution buffer, incubated for 5 min, and centrifuged at 16,000 rpm for 1 min.

### DNA amplification

The amplification process occurred in four stages in the AB-PCR system. PCR conditions were as follows: pre-denaturation, denaturation, annealing, elongation, and final elongation of the DNA molecule. The initial denaturation stage was conducted for one cycle at 95°C for 1 min. The second stage was performed for 35 cycles, with each cycle consisting of denaturation at 95°C for 15 sec. Annealing was performed at 60°C for 10 sec, and extension was at 72°C for 10 sec.

### Sequencing

The amplicon target was subjected to Sanger sequencing using Macrogen Analysis services in South Korea. Sequencing was used to elucidate the nucleotide sequence of the DNA molecule.

### Data analysis

#### SNP variations of the CSN1S1 gene

Identification of the base mutations of the CSN1S1 gene resulting from the sequencing process was analyzed using the MEGA10 and PopGen32 applications to determine the existence of base mutations and DNA polymorphisms.

#### Association of genotypes and milk components

The association of genotype variants of the CSN1S1 gene with the content or yield (gm/day) of milk protein and other MCs was analyzed utilizing a General Linear Model (GLM) for unbalanced data. The fixed effects of genotype and non-genetic factors (lactation stage, lactation period, kidding season, and kidding year) were simultaneously incorporated into the GLM models. Data were analyzed using the SAS/STAT^®^ software (SAS Institute Inc., Cary, NC, USA, v9.1). The mathematical model is as follows:

Y_ijklmn_ = µ + G_i_ + T_j_ + P_k_ + S_l_ + Y_m_ + e_ijklmn_

**Table:** 

Y_ijklmn_	=	content (%) or yield (gm/day) of each milk component.
µ	=	overall mean.
G_i_	=	effect of the i^th^ genotype of individual SNPs in the CSN1S1 gene.
T_j_	=	effects of the j^th^ lactation length (1-2, 3-4, and ≥5).
P_k_	=	effect of the k^th^ lactation period (1, 2, and ≥3).
S_l_	=	effect of the l^th^ kidding season (January-June, July-December).
Y_m_	=	effect of the m^th^ kidding year (2021, 2022).
*ε*_ ijklmn_	=	random error.

Statistically significant differences between the means of content (%) or yield (gm/day) of milk protein and other MCs among subclasses were evaluated using Duncan’s multiple range test at *p *< 0.05.

## Results and Discussion

### Description of milk components

Statistical analyses of the mean, standard error (SE), coefficient of variation (CV), minimum (Min), and maximum (Max.) values for both the content and yield (gm/day) of MCs and daily milk yield (DMY) (ml/day) of the local PE cross-dairy goats are presented in Table 1. The average contents of protein, fat, lactose, SNF, and total solids based on the results of the DMY test were consistent with the content of goat MCs reported [7,14,12], higher than those reported by [17], but slightly lower than those observed by [6] and [11].

The yields (gm/day) of milk protein or other MCs were calculated based on the formula presented in the research method. A slight variation in the values was attributable to the influence of DMY and its specific gravity. The mean content and yield values of MCs were within acceptable ranges (CV = 5.79%–8.29%), whereas those for fat and total solids were relatively high (CV = 11.37%–12.10%), and the DMY exhibited a notably high coefficient of variation (CV = 51.18%). The high CV value for total solids was likely attributable to variations in milk fat content. The significant CV for DMYs suggests a substantial influence of environmental and genetic factors on milk quantity and quality.

**Table 1.** Statistical description of content (%) and yield (gm/day) of milk components and daily milk yield (ml/day).

Variable	*N*	Mean ± SE		Min.-Max.	CV	Mean ± SE		Min.-Max.	CV
		Content (%)			%	Yield (gm/day)			%
Protein	65	3.73 ± 0.03	3.40–4.50		5.79	37.32 ± 0.27	34.00–45.00		5.79
Fat	65	4.51 ± 0.10	3.67–6.20		12.10	45.13 ± 1.01	36.70–62.00		11.20
SNF	65	7.70 ± 0.06	6.66–9.57		6.59	76.97 ± 0.63	66.60–95.70		6.59
Lactose	65	3.61 ± 0.04	3.20–4.80		8.29	36.11 ± 0.37	32.00–48.00		8.29
Total solid	65	12.46 ± 0.18	10.17–16.20		11.37	124.6 ± 1.76	101.7–162.0		11.43
DMY (ml/day)	65	906.1 ± 73.1	500–2350		51.18	-	-		-

N: number of observations; SE: standard error; CV: coefficient of variation; Min.-Max.: minimum to maximum, SNF: solid non-fat; DMY: daily milk yield.

**Figure 1.**
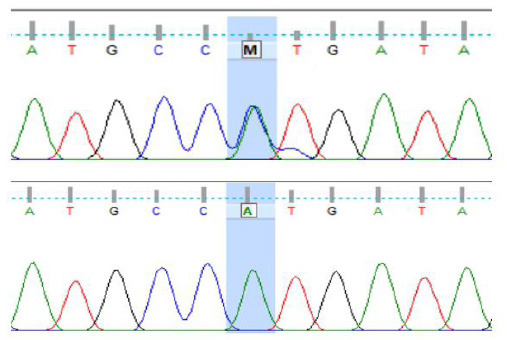
Visualization of the Sanger sequencing bands at one of the mutation loci (SNP g.10180 A > C).

### SNPs of the CSN1S1 gene

The amplicon product of the base fragment target of the CSN1S1 gene, flanked by the forward primer at positions 11,761–11,780 and the reverse primer at positions 12,375–12,394, yielded a 634 bp sequence. This sequence encompasses DNA fragments within intron 11, exon 12, and partial intron 12. Sanger sequencing of the fragment target revealed nucleotides in the form of a chromatogram. The DNA sequences, representing adenine (A) in green, guanine (G) in black, cytosine (C) in blue, and thymine (T) in red, demonstrate the presence of nucleotides (Fig. 1). To identify potential base mutations, the DNA sequences were compared with the reference bases (AJ504710.2) and subjected to Finch TV analysis. This study focused on point-based mutations, specifically SNPs in the CSN1S1 g.10094–g.10200 (368 bp). Visualization of the identified SNPs is illustrated by the presence of such as the SNP g.10180 A > C (Fig. 1).

Following sequencing analysis of the CSN1S1 fragment g.11,761 to g.12,394, 15 SNPs were identified in the amplicon product (634 bp). However, this investigation primarily focused on point-based mutations or SNPs along CSN1S1 g.10094–g.10200 (368 bp) in intron 11 of the CSN1S1 gene. Eight SNPs and one insertion mutation in the form of Ins AGAA g.10090–g.10093 were observed. The SNPs were identified regarding the locus location in the order: SNP g.10098_C > G, SNP g.10112_C > T, SNP g.10129_C > A, SNP g.10177_G > A, SNP g.10180_A > C, SNP g.10181_T > A, SNP g.10188_T > C, and SNP g.10197_C > T (Table 2). The numerical designations represent specific positions within the CSN1S1 gene. For instance, SNP g.10098_C > G indicates a base substitution from cytosine (C) to guanine (G) at the 10098th base pair from the initiation of the CSN1S1 gene.

**Table 2:** Identification of SNPs at the base fragment CSN1S1 g.10094–g.10200.

Locus of the CSN1S1 gene	Genotype frequency			Allele frequency	
g.10098­­_C > G (SNP-1)	CC	CG	GG	C	G
	0.77	0.23	-	0.89	0.11
g.10112_C > T (SNP-2)	CC	TC	TT	C	T
	0.85	0.15	-	0.92	0.08
g.10128_C > A (SNP-3)	AA	AC	CC	C	A
	-	0.05	0.95	0.98	0.02
g.10177_G > A (SNP-4)	AA	AG	GG	G	A
	0.18	-	0.82	0.82	0.18
g.10180_C > A (SNP-5)	AA	AC	CC	C	A
	0.47	0.53	-	0.27	0.73
g.10181_T > A (SNP-6)	AA	AT	TT	T	A
	0.15	0.03	0.82	0.83	0.17
g.10188_T > C (SNP-7)	CC	CT	TT	T	C
	0.20	-	0.80	0.80	0.20
g.10197_T > C (SNP-8)	CC	CT	TT	T	C
	0.20	-	0.80	0.80	0.20

SNP: Single Nucleotide Polymorphism.

These findings align with the confirmed structure of CSN1S1 as a highly polymorphic gene, exhibiting numerous synonymous and non-synonymous mutations [6,10,11,16]. The eight SNPs identified in this study will serve as valuable molecular maps for exploring their potential application as selection biomarkers to enhance milk quality traits, including protein content and other MCs of local PE cross-dairy goats.

### Allele and genotype frequencies

The genotype and allele frequencies of each SNP of the CSN1S1 gene are presented in Table 2. Among the eight SNPs found in this study, seven SNPs (SNP g.10098_C > G, SNP g.10112_C > T, SNP g.10129_C > A, SNP g.10177_G > A, SNP g.10181_T > A, SNP g.10188_T > C, and SNP g.10197_C > T) exhibited a predominance of homozygous genotype references with genotype frequencies ranging from 0.77 to 0.95, resulting in high frequencies of the reference allele (0.73–0.98) and the mutant allele (0.08–0.27). In contrast, the remaining SNP g.10180_C > A was the sole SNP with a dominant heterozygous genotype mutant, which resulted in a higher frequency of the A allele mutant compared to the C allele reference (0.73 *vs.* 0.27).

All the SNPs were classified as polymorphic due to their multiple alleles, each with a frequency exceeding 0.01 10,11. Polymorphic SNPs are present in intron 11 of the CSN1S1 gene. These findings confirmed the statement that the CSN1S1 gene exhibits high polymorphism in caprine, characterized by numerous synonymous and non-synonymous mutations [10]. Current research has indicated that a minimum of 20 genetic variants have been identified for alpha-S1 casein (CSN1S1), eight for beta casein (CSN2), 14 for alpha-S2 casein (CSN1S2), and 24 for kappa casein (CSN3) in diverse goat breeds [2].

The seven SNPs in this study predominantly exhibited reference or non-mutant alleles. There was also a considerable frequency range between the reference and mutant alleles for all SNPs. The reference allele occurs more frequently than the mutant allele [6,10,11]. A preliminary study on the SNP g.12164_G > A at exon 12 of CSN1S1 revealed a higher frequency of the reference allele (G) than the mutant allele (A) in the PE crossbred goats, with PE as the local parental breed and Saanen as the exotic parental breed [16]. High genetic polymorphisms in the CSN1S1 gene were also observed in exon 12 [10], introns 11-13 [18], and introns 11-13 [6,13] from various native and exotic (European) goat breeds.

Variations in SNPs frequently manifest in a gene or distinct genes across breeds within species [11]. These genetic variants might probably arise from different breeding practices and adaptive processes [11,13]. Conversely, multiple studies have identified several SNPs in the CSN1S1 gene exhibiting monomorphic conditions, characterized by the presence of only one allele or an allele frequency below 0.01 [10,11,19].

The genetic diversity of single genes or cluster genes can be attributed to multiple factors, including mating patterns, mutations, genetic drift, and artificial selection, which may vary between goat populations or breeds [11,16,19]. The presence of polymorphic SNPs in the CSN1S1 g.10094–g.10200 suggests that the observed crossbred goats, resulting from naturally random mating with sufficient Saanen bucks, have not been subjected to specific selection for enhanced milk quality.

### Effect of the SNPs on milk components

Information regarding the effects of genetic variants of the CSN1S1 gene on milk quality and MCs has been extensively documented in both exotic and non-exotic caprine breeds. Different breeds may exhibit distinct genetic responses to the existence of SNPs and their interactions within genes or different genes when examining the SNP effects as biomarker selection in improving the considered trait [11]. So, this study aimed to determine the effect of SNPs along CSN1S1 g.10094–g.10200 by examining the association of each SNP with the contents and yield (mg/day) of milk protein and other MCs in the local PE crossbred goats. Following the identification of the beneficial alleles of the identified SNPs, it is anticipated that molecular selection will facilitate the enhancement of quality and MC traits in these candidate new crossbred goats.

Milk components are quantitative traits that are influenced by genetic and non-genetic factors associated with lactation traits. Multiple factors determine the composition, including protein, fat, lactose, SNP, total solids, SNP, and other MCs. Several environmental factors can influence MCs and quality, such as milking frequency, lactation stage, lactation period, parity (age), management, season of lactation, and year of lactation [2].

Several non-genetic factors, particularly the season and year of birth, consistently influenced the content and yield of milk protein and other MCs (*p *< 0.05, *p *< 0.01) based on the preliminary analysis results in the crossbred goats. The association study was conducted by applying the GLM to unbalanced data, simultaneously considering the influence of each SNP and differences in the lactation stage, lactation period, kidding season, and kidding year of lactating does. This approach aims to eliminate the influence of non-genetic factors when examining the effect of each SNP on milk protein and each MC [2,19].

The effects of each SNP on the content of MCs and DMY (ml/day) are presented in Table 3, while those effects on the yields (gm/day) of MCs are shown in Table 4. As demonstrated in Table 3, SNP g.10181_T > A significantly influenced the contents of protein, fat, lactose, and total solids (*p *< 0.05), but not the SNF content (*p *> 0.05). However, all MC contents were not significantly affected by the remaining seven SNPs (*p *> 0.05). Further, as presented in Table 4, the yields (gm/day) of protein, fat, lactose, SNF, and total solids were significantly influenced (*p *< 0.05) by the SNP g.10098 C > G. The remaining seven SNPs, however, exhibited no significant (*p *> 0.05) effects on the yields of any MCs. Moreover, SNP g.10098_C > G significantly impacted (*p *< 0.05) DMY (ml/day) (Table 3).

**Table 3.** LSM and SE of the contents (%) of the milk components and daily milk yield (ml/day) based on the genotype of the CSN1S1 gene.

Locus of CSN1S1 gene	Geno-type	*N*	Protein	Fat	Lactose	SNF	Total Solid	Milk yield
g.10098 C > G	CC	51	3.73 ± 0.05	4.53 ± 0.18	3.58 ± 0.06	7.80 ± 0.12	12.82 ± 0.31	722 ± 67^a^
	GC	15	3.75 ± 0.06	4.66 ± 0.20	3.59 ± 0.07	7.87 ± 0.14	12.87 ± 0.36	648 ± 106^b^
g.10112 C > T	CC	56	3.71 ± 0.05	4.59 ± 0.17	3.57 ± 0.06	7.80 ± 0.11	12.84 ± 0.29	714 ± 64
	CT	10	3.81 ± 0.07	4.54 ± 0.24	3.64 ± 0.09	7.93 ± 0.17	12.85 ± 0.42	610 ± 117
g.10128 C > A	CA	3	3.71 ± 0.14	4.46 ± 0.46	3.58 ± 0.16	7.73 ± 0.31	12.59 ± 0.80	803 ± 101
	CC	63	3.74 ± 0.05	4.60 ± 0.16	3.58 ± 0.06	7.84 ± 0.11	12.87 ± 0.29	675 ± 59
g.10177 G > A	AA	12	3.71 ± 0.07	4.44 ± 0.23	3.54 ± 0.08	7.87 ± 0.16	12.68 ± 0.40	727 ± 116
	GG	54	3.75 ± 0.05	4.65 ± 0.18	3.61 ± 0.06	7.80 ± 0.12	12.93 ± 0.31	679 ± 66
g.10180 C > A	AC	35	3.72 ± 0.05	4.51 ± 0.18	3.56 ± 0.06	7.76 ± 0.12	12.65 ± 0.30	752 ± 74
	AA	31	3.76 ± 0.06	4.68 ± 0.19	3.61 ± 0.07	7.91 ± 0.13	13.11 ± 0.33	620 ± 84
g.10181 T > A	AA	10	3.60 ± 0.07^a^	4.18 ± 0.23^a^	3.40 ± 0.08^a^	7.60 ± 0.16	12.04 ± 0.40^a^	779 ± 114
	AT	2	3.85 ± 0.15^b^	5.18 ± 0.49^b^	3.81 ± 0.17^b^	8.13 ± 0.34	13.76 ± 0.83^b^	722 ± 67^a^
	TT	54	3.78 ± 0.05^a^	4.68 ± 0.17^a^	3.63 ± 0.06^a^	7.89 ± 0.12	13.07 ± 0.30^a^	648 ± 106^b^
g.10188 T > C	CC	13	3.68 ± 0.07	4.42 ± 0.22	3.50 ± 0.08	7.76 ± 0.15	12.51 ± 0.38	714 ± 64
	TT	53	3.76 ± 0.05	4.66 ± 0.17	3.63 ± 0.06	7.86 ± 0.12	13.01 ± 0.30	610 ± 117
g.10197 T > C	CC	13	3.68 ± 0.07	04.42 ± 0.22	3.50 ± 0.08	7.76 ± 0.15	12.51 ± 0.39	803 ± 101
	TT	53	3.76 ± 0.05	4.66 ± 0.17	3.63 ± 0.06	7.86 ± 0.12	13.01 ± 0.30	675 ± 59

LSM: least-squares mean; SE: standard error; N: number of observations; SNF: solid not fat; Different letters between means in the same column indicated statistically significant differences (*p *< 0.05).

**Table 4.** LSM and SE of the yields (gm/day) of milk components based on the genotype of the CSN1S1 gene.

Locus of CSN1S1 gene	Geno-type	N	Protein	Fat	Lactose	SNF	Total Solid
g.10098 C >G	CC	51	23.09 ± 4.16^a^	26.86 ± 4.97^a^	22.36 ± 3.97^a^	48.10 ± 8.43^a^	65.98 ± 14.02^a^
	GC	15	16.95 ± 4.89^b^	19.23 ± 5.85^b^	16.26 ± 4.65^b^	35.43 ± 9.91^b^	47.35 ± 15.61^b^
g.10112 C > T	CC	56	21.91 ± 3.89	25.43 ± 4.65	21.15 ± 3.71	45.74 ± 7.89	61.61 ± 13.08
	CT	10	16.74 ± 5.71	18.84 ± 6.83	16.21 ± 5.45	34.72 ± 11.58	46.69 ± 18.52
g.10128 C > A	CA	3	26.50 ± 10.83	27.75 ± 12.99	25.92 ± 10.33	54.68 ± 21.97	73.09 ± 30.68
	CC	63	20.14 ± 3.88	23.57 ± 4.66	19.41 ± 3.71	42.06 ± 7.88	56.15 ± 13.33
g.10177 G > A	AA	12	21.74 ± 5.43	25.07 ± 6.51	20.73 ± 5.19	46.24 ± 11.20	64.61 ± 17.96
	GG	54	20.30 ± 4.22	23.45 ± 5.05	19.74 ± 4.03	41.87 ± 8.55	55.94 ± 13.63
g.10180 C > A	AC	35	23.68 ± 4.09	27.10 ± 4.91	22.82 ± 3.90	48.87 ± 8.31	51.34 ± 14.89
	AA	31	16.70 ± 4.48	19.62 ± 5.39	16.20 ± 4.28	35.53 ± 9.11	64.33 ± 14.21
g.10181 T > A	AA	10	23.30 ± 5.77	25.56 ± 6.86	22.05 ± 5.51	49.46 ± 11.68	69.87 ± 18.97
	AT	2	28.54 ± 11.93	40.14 ± 14.19	28.36 ± 11.39	59.63 ± 24.15	84.35 ± 35.74
	TT	54	18.75 ± 4.23	21.33 ± 5.03	18.20 ± 4.04	38.72 ± 8.56	51.07 ± 13.76
g.10188 T > C	CC	13	21.31 ± 5.32	26.63 ± 6.37	20.33 ± 5.08	45.24 ± 10.79	63.03 ± 17.70
	TT	53	20.52 ± 4.16	23.68 ± 4.99	19.94 ± 3.97	42.39 ± 8.44	56.63 ± 13.53
g.10197 T > C	CC	13	21.31 ± 5.32	24.63 ± 6.37	20.33 ± 5.08	45.24 ± 10.79	63.03 ± 17.70
	TT	53	20.52 ± 4.16	23.68 ± 4.99	19.94 ± 3.97	42.39 ± 8.44	56.63 ± 13.53

LSM: least-squares mean; SE: standard error; N: number of observations; SNF: solid not fat; Different letters between means in the same column indicated statistically significant differences (*p *< 0.05).

Polymorphism SNP g.10181_T > A caused a significant difference (*p *< 0.05) in the content of milk protein and other MCs (Table 3). The milk protein content of the heterozygous AT genotype was higher compared to those of the TT genotype does and the AA genotype ones, i.e., 1.85% and 6.94%, respectively. Likewise, SNP g.10181_T > A regarding the AT does, compared to the AA and TT does, consistently generate more fat, lactose, and total solids. Further, the variant genetics of the SNP g.10098_C > G showed approximately 36.22% more milk protein was generated by the CC genotype than the GC one.

While polymorphism SNP g.10098_C > G also gave a significant impact (*p *< 0.05) on the yields (gm/day) of other MCs, from the CC does to the GC ones, consistently favoring the production of fat, lactose, and SNF, with the benefits of 39.58%, 37.52%, and 35.76%, respectively. Further, this SNP g.10098_C > G consistently affected DMY (ml/day), of which the CC genotype was beneficial by 11.4% compared to the GC one (Table 3). Although this SNP was located in a non-coding region, it was able to interact with SNPs in other coding regions (exons) to influence the levels of gene expression and, thus, the amount of RNA and protein, as reported from the previous studies [10,11,19].

The effects of CSN1S1 polymorphism on milk quality and MCs have been reported in many dairy goats. Previous research [11] demonstrated a significant (*p *< 0.01) effect of the SNPs rs664719033 and rs155505528 of the CSN1S1 gene on milk protein and fat content [17]. The CSN1S1 genotype accounted for 8.3% to 9.2% of the variation in protein and fat content. By considering the findings of our current study, it can be concluded that the two SNPs may serve as valuable genetic markers of selection for improving milk quality, specifically the SNP g.10098_C > G for the MC yields and DMY and the SNP g.10181_T > A for the MC content in the local PE cross-dairy goat.

This study offers valuable insights into genetic diversity and the potential of two SNPs as marker selection for enhancing MCs in the local PE cross-dairy goats. However, the limited sample size of goat samples, DMYs, and phenotypic MCs could bias the examination of SNP effects on the CSN1S1 g.10094-g.10200. To alleviate this, we employed stringent statistical tests suitable for a small sample size. Despite these limitations, our findings are significant and underscore the need for further research. Further investigations utilizing larger sample sizes, comprehensive analyses of CSN1S1 SNPs, and phenotypic data are necessary to validate and expand upon our findings. Such studies will provide a more profound understanding of SNPs as variant genes of the CSN1S1 gene and their implications to be used as biomarker selection for enhancing milk protein and other MCs. This knowledge is imperative for developing breeding strategies to enhance milk quality and components in local PE cross-dairy goats, which constitute a critical genetic resource for the community’s milk and dairy industries.

## Conclusion

Substantial genetic variation was observed in the CSN1S1 gene at the base fragment g.10094–g.10200, with the highest frequency of dominant SNPs (seven SNPs) exhibiting the homozygous genotype reference. Two potential SNPs could be considered as genetic markers for selection to enhance milk quality: SNP g.10098_C > G for the yield of milk components and milk production and SNP g.10181 T > A for the content of milk components. This knowledge is essential for developing breeding strategies to enhance milk quality and components in local PE cross-dairy goats and parentally local PE goats, which constitutes a critical genetic resource for the community’s milk and dairy industries.

## List of abbreviations

AA, AT, TT, CC, CG: genotype; CSN1S1, casein alpha S1; CV, coefficient of variation; d: day; DNA, deoxyribonucleic acid; GLM, general linear model; gm, gram; IRIAP, indonesian research institute for animal production; Max, maximum; MCs, milk components; Min, minimum; min, minute; µl, microliter; ml, milliliter; N, number of observations; PE, peranakan etawa; RBC, red blood cell; RNA, ribonucleic acid; RPM, revolutions per minute; SAS, statistical analysis software; SE, standard error; sec, second; SNF, solid non-fat; SNP, single-nucleotide polymorphism; TDMY, test day milk yield; TS, total solid.

## References

[1] Clark S, García MBC (2017). A 100-year review: advances in goat milk research 1. J Dairy Sci.

[2] Rahmatalla SA, Arends D, Brockmann GA (2022). Review: genetic and protein variants of milk caseins in goats. Front Genet.

[3] Saikia D, Hassan MHI, Walia A (2022). Review: goat milk and its nutraceutical properties. Int J Appl Poultry Res.

[4] Magan JB, O’Callaghan TF, Kelly AL, McCarth NA (2021). The composition and function of milk and its derivatives derived from cows fed on pasture or concentrate-based diets. Compr Rev Food Sci Food Saf.

[5] Hassanin A, Osman AA, Atallah A, El-Saadony O, Abdelnour MT, Awad SA (2022). Phylogenetic comparative analysis: chemical and biological features of caseins (alpha-S-1, alpha-S-2, beta- and kappa-) in domestic dairy animals. Front Vet Sci.

[6] Johansson M, Lundh A, Johansson AM (2023). Relation between *α*S1-casein, genotype, and quality traits of milk from Swedish dairy goats. J Dairy Sci.

[7] Chauhan S, Powar P, Mehra R (2021). A review on nutritional advantages and nutraceutical properties of cow and goat milk. Int J Appl Res.

[8] Caboni P, Murgia A, Porcu A, Demur M, Pulina G, Nudda A (2016). Gas chromatography-mass spectrometry metabolomics of goat milk with different polymorphism at the S1-casein genotype locus. J Dairy Sci.

[9] Abousoliman MMI, Reyer H, Oster M, Muráni E, Abdel-Salam IMM, Wimmers K (2020). Analysis of candidate genes for growth and milk performance traits in the Egyptian Barki sheep. Animals.

[10] Rahmatalla SA, Arends D, Ahmed AS, Hassan LMA, Krebs S, Reissmann1 M (2021). Capture sequencing to explore and map rare casein variants in goats. Front Genet.

[11] Dettori ML, Pazzola M, Noce A, Landi V, Vacca GM (2024). Variations in casein genes are associated with milk protein and fat contents in Sarda goats (*Capra hircus*), with an important role of CSN1S2 for milk yield. Animals.

[12] Khaldi Z, Nafti M, Jilani MT, Souid S (2023). Effect of kappa casein and beta lactoglobulin genetic variants on milk composition traits in Tunisian oasis autochthonous goats. J Adv Vet Res.

[13] Gašper J, Miluchová M, Gábor M (2023). Analysis of the genetic structure of Slovak white shorthaired goat breed using CSN1S1 gene. J Cent Eur Agric.

[14] Nayik GA, Jagdale YD, Gaikwad SA, Devkatte AN, Dar AH, Ansari MJ (2022). Nutritional profile, processing and potential products: a comparative review of goat milk. Dairy.

[15] Anggraeni A, Saputra F, Kumalawati DS, Sumantri C (2020). Effect of litter size, kidding age, mating weight, and kidding weight on partial cumulative milk yields in G1 Sapera goat. IOP Conf Ser: Earth Environ Sci.

[16] Anggraeni A, Syifa L, Sari OK, Ishak ABL (0200). Polymorphism of CSN1S1 (g.12164G>A) and CSN2 (g .8913C>A ) genes in pure and cross dairy goats, BIO Web Conf. 2021;.

[17] Pizarro MG, Landi V, González FJN, León JM, Delgado IV (2019). Non-parametric analysis of the effects of *α*S1-casein genotype and parturition non-genetic factors on milk yield and composition in Murciano-Granadina goats. Ital J Anim Sci.

[18] Widodo HS, Murti TW, Agus A, Pertiwiningrum A (2023). Identification of CSN1S1 gene variations between dairy goat breeds and its influence on milk protein fractions in Indonesia. Adv Anim Vet Sci.

[19] Gipson TA (2019). Recent advances in breeding and genetics for dairy goats. Asian-Australas J Anim Sci.

